# Response to: Limitations of the p16-3MR mouse model for detecting and eliminating senescent cells

**DOI:** 10.1038/s44319-026-00801-9

**Published:** 2026-05-28

**Authors:** Boshi Wang, Jamil Nehme, Marco Demaria

**Affiliations:** European Research Institute for the Biology of Ageing (ERIBA); University of Groningen (RUG); University Medical Center Groningen (UMCG), Groningen, The Netherlands

**Keywords:** Methods & Resources, Molecular Biology of Disease

## Abstract

Response to: Limitations of the p16-3MR Mouse Model for Detecting and Eliminating Senescent Cells by Hori et al?

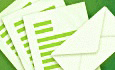

Since its development led by the laboratory of Judith Campisi and colleagues, the p16-3MR mouse model has been widely distributed and adopted across laboratories worldwide to visualize and conditionally ablate p16^Ink4a^-expressing cells in vivo. Numerous studies performed outside the originating laboratory have reported consistent and biologically meaningful phenotypes across contexts, including tissue repair, fibrosis, cancer, therapy response, and organismal aging, typically validated using senescence markers independent of the transgene. While we were involved in the initial publication describing the application of this model, our perspective here reflects its extensive subsequent use across multiple independent laboratories and experimental contexts.

Hori et al (2026) recently concluded that the p16-3MR construct is non-functional, a position that is not supported by the broader body of independent experimental evidence. Importantly, the conclusion that all three components of the transgene are non-functional represents a generalization from a specific experimental configuration that does not account for variability in experimental context, detection sensitivity, and biological setting, including the use of non-littermate controls. Here, we provide a focused technical clarification of the issues raised, while new experimental data relevant to these clarifications are made publicly available as a preprint (Wang et al, 2026).

## Bioluminescence signal specificity and background

Hori et al (2026) argue that bioluminescent signals observed in p16-3MR mice largely reflect auto-oxidation of coelenterazine-h (CTZ-h), and further assert that signals below a fixed photon-count threshold (600 counts) should be considered background. This interpretation does not account for key technical variables.

There is no universal photon-count cutoff that defines biological background across imaging platforms. Absolute counts depend on detector sensitivity, exposure time, binning, optics, and instrument-specific scaling (IVIS, LAGO, etc.). The biologically meaningful criterion is signal-to-background ratio and reproducible modulation across biological conditions, not adherence to a fixed numerical threshold. Indeed, chemical auto-oxidation of CTZ-h produces a relatively static background, whereas p16-3MR signals reported across multiple studies show dynamic regulation, increasing with aging, injury, or genotoxic stress and decreasing following ganciclovir treatment, which is not readily explained by substrate chemistry alone. Reproducible biological modulation of signal across independent studies is therefore not consistent with a purely chemical background origin.

The observation of comparable signals between wild-type and transgenic animals under specific imaging conditions, as reported by Hori et al (2026), is consistent with measurements performed near the detection limits of the system. Under such conditions, a biological signal may not be resolvable from the background, particularly when reporter expression is low or spatially restricted. Thus, the absence of separation between genotypes under low-sensitivity conditions does not necessarily indicate the absence of a biological signal.

It is also important to note that absolute photon counts reported across studies cannot be interpreted independently of imaging configuration and normalization strategies. Cross-study comparison of raw counts without harmonization of acquisition parameters is inherently unreliable and may lead to misclassification of biologically meaningful signal as background.

Spectral filtering can be used as an orthogonal validation of signal specificity (Wang et al, 2026), but it is not a requirement for p16-3MR functionality.

## Pigmentation, hair cycling, and shaving

Pigmentation and hair cycling are acknowledged limitations of all optical imaging approaches. While shaving or albino backgrounds can improve sensitivity, pigmentation does not preclude detection when p16+ cells accumulate in sufficient abundance, as demonstrated in independent studies using non-shaved black-furred animals.

Importantly, hair removal itself is not biologically neutral. Shaving, depilation, or tape-stripping induces micro-injury and local inflammation in murine skin, a context in which p16^Ink4a^ engagement is well documented (Gao et al, 2013; Hsu et al, 2018; He et al, 2022). Thus, shaving may introduce a biological confound in certain contexts, rather than acting purely as an optical refinement. Shaving should therefore be considered a context-dependent, but not absolute, refinement.

## Reproducibility of age- and stress-associated induction

Multiple independent studies have reported age- and stress-associated activation of p16-3MR readouts or downstream phenotypes. Notably, similar age-associated p16-driven reporter activation has also been reported using an independent p16-luciferase model developed by the Sharpless and the Hori group itself (Liu et al, 2019; Kawamoto et al, 2023), underscoring that the biological phenomenon is not model-specific.

Failure to reproduce specific phenotypes under a given experimental configuration does not, in itself, demonstrate a lack of model functionality, particularly when multiple independent studies have reported context-dependent activation using orthogonal validation strategies.

## Functionality of the HSV-tk/ganciclovir system

In vitro functionality of the HSV-tk cassette in p16-3MR cells was demonstrated in the original report and independently reproduced (Baker et al, 2016). In vivo, partial clearance (50–70%) is expected for HSV-tk systems due to tissue penetration limits, heterogeneous expression, and bystander effects. Importantly, the absence of detectable cell elimination under a given dosing or experimental paradigm does not demonstrate the absence of HSV-tk functionality, but may reflect insufficient transgene expression, pharmacokinetic constraints, or biological resistance of specific senescent cell populations.

Crucially, most published p16-3MR studies validated senescent-cell reduction using orthogonal markers independent of the transgene (SA-β-gal, p16, p21, γH2AX, LaminB1, SASP factors), arguing against nonspecific drug effects as the sole explanation for observed phenotypes. These convergent observations across independent markers and biological systems are not consistent with a purely off-target pharmacological effect.

## Interpretation of ganciclovir effects

Ganciclovir has known ancillary biological effects under specific dosing regimens. HSV-tk–independent interferon responses have been reported (Mathur et al, 2017), but only at substantially higher doses and longer treatment durations than those used in standard p16-3MR protocols (Demaria et al, 2014). We agree that ganciclovir can exert context-dependent, HSV-tk–independent biological effects, particularly in immune cells.

However, this does not preclude its use as a conditional ablation tool when interpreted alongside orthogonal senescence markers and appropriate experimental controls. Recent work highlighting immune modulation following p16^+^-cell clearance underscores biological interconnectedness between senescence/immunity rather than model failure (Omori et al, 2020; Zhao et al, 2024).

## Environmental and facility variables

Differences in housing conditions, microbiota composition, pathogen exposure, diet, and genetic background are well established to influence aging trajectories, senescence burden, and reporter output. In this context, comparisons between transgenic animals and commercially sourced wild-type controls, rather than littermate controls, introduce additional uncontrolled genetic and environmental variability that can substantially affect baseline p16 expression and signal detection.

Under such conditions, the absence of clear separation between genotypes cannot be unambiguously interpreted as the absence of reporter activity, as it may reflect differences in genetic background, environmental exposure, or overall senescent cell burden rather than transgene performance.

Absolute signal intensity is therefore not directly comparable across facilities or imaging systems and should be interpreted within the same experimental and biological context.

## Conclusion

The p16-3MR model remains a functional and reproducible tool for studying p16-positive senescent cells in vivo when interpreted within an appropriate experimental and technical context.

Apparent discrepancies in signal detection or clearance across studies are most likely explained by differences in experimental design, imaging implementation, detection sensitivity, and biological context, rather than failure of the transgene.

The central question is therefore not whether the p16-3MR system produces high-intensity reporter signals under all conditions, but whether it can reliably capture biologically meaningful variation in p16+ cell burden when applied within an appropriate experimental framework.

Importantly, the biological phenomena captured by this model have been independently observed across multiple experimental systems, supporting their robustness beyond any single reporter construct.

## Supplementary information

Peer Review File
