# Supplementary material for: Response to: Limitations of the p16-3MR mouse model for detecting and eliminating senescent cells
Source: EMBO Rep. 2026 May 28;27(13):3535–7. doi: 10.1038/s44319-026-00801-9 (PMC13354777; doi:10.1038/s44319-026-00801-9)
Supplement: Supplementary file 1 — Peer Review File [file 44319_2026_801_MOESM1_ESM.pdf]

EMBO reports

Response to: Limitations of the p16-3MR Mouse Model for Detecting and Eliminating Senescent Cells

Boshi Wang, Jamil Nehme, and Marco Demaria

Corresponding author(s): Marco Demaria (m.demaria@umcg.nl) , Boshi Wang (b.wang01@umcg.nl)

Review Timeline:

Submission Date:	27th Oct 25
Editorial Decision:	7th Dec 25
Revision Received:	2nd Feb 26
Editorial Decision:	25th Mar 26
Revision Received:	17th Apr 26
Editorial Decision:	26th Apr 26
Revision Received:	4th May 26
Accepted:	7th May 26

Editor: Bernd Pulverer

Transaction Report:

(Note: With the exception of the correction of typographical or spelling errors that could be a source of ambiguity, letters and reports are not edited. Depending on transfer agreements, referee reports obtained elsewhere may or may not be included in this compilation. Referee reports are anonymous unless the Referee chooses to sign their reports.)

1st Editorial Decision 7th Dec 2025

Dear Dr. Demaria

Thank you for the submission of your response to the paper by Hara and colleagues to EMBO reports. We have now received two referee reports as well as short statement form a third referee.

As you will see, the referees are not supportive of publication of the data you submitted in the absence of definitive controls.

In summary, the referees note:
ref1: 'the data presented do not adequately refute the findings of the Hara Lab. The principal concern is the consistent lack of adequate wild-type controls. Without rigorous comparison to properly matched non-transgenic mice, conclusions regarding bioluminescence, reporter specificity, or the effects of GCV and doxorubicin remain insufficiently supported.
specifically,'
(i) In Figure 1, no wild-type controls were included.
(ii) The authors do not demonstrate that reductions in bioluminescent signal following GCV treatment are caused by ablation of senescent cells.

ref 2:
(i) all the results shown in Figure 1 lack appropriate age-matched WT controls. Such controls, including multiple animals, ought to be included for all experiments presented.
(ii) a concern is the organ source of the bioluminescent signal. Whole-body images indicate a maximal signal in the abdominal region, potentially consistent with senescent cells in adipose tissue. Yet, upon examination of organs from aged animals, the lungs and spleen exhibit very high signals-something not apparent in the whole-body imaging. This discrepancy is concerning. As previously noted, age-matched WT controls are absent from this analysis, rendering the interpretation of these results rather challenging.
(iii) The referee fails to see a need for table 1 out.

Ref 3 also comments on likely technical deficiencies of the model, but did not specify details.

While we therefore cannot publish the current dataset, I would nonetheless like to invite you to revise your response to Hara and colleagues to a short textual statement. If you wish to refer to the preliminary data you generated, one possibility would be to post it as a preprint and to cite that in your short response.
Please address all referee concerns in a complete point-by-point response. Acceptance of the manuscript will depend on a positive outcome of a second round of review.

We realize that it is difficult to revise to a specific deadline. In the interest of protecting the conceptual advance provided by the work, we recommend a revision within 2 months (9th Feb 2026).

General notes (please ignore specifics that are not applicable):
We perform an initial quality control of all revised manuscripts before re-review.
When submitting your revised manuscript, please review the submission guidelines (https://link.springer.com/journal/44319/submission-guidelines#cms-Revised-submissions) and carefully review the instructions that follow below. Failure to include requested items will delay the evaluation of your revision.
1) a .docx formatted version of the manuscript text (including legends for main figures, EV figures and tables). Please make sure that the changes are highlighted to be clearly visible.
2) individual production quality figure files as .eps, .tif, .jpg (one file per figure). See hhttps://media.springernature.com/original/springer-cms/rest/v1/content/27825798/data/v1 for more info on how to prepare your figures.
3) We replaced Supplementary Information with Expanded View (EV) Figures and Tables that are collapsible/expandable online. A maximum of 5 EV Figures can be typeset. EV Figures should be cited as 'Figure EV1, Figure EV2" etc... in the text and their respective legends should be included in the main text after the legends of regular figures.

- For the figures that you do NOT wish to display as Expanded View figures, they should be bundled together with their legends in a single PDF file called *Appendix*, which should start with a short Table of Content. Appendix figures should be referred to in the main text as: "Appendix Figure S1, Appendix Figure S2" etc. See 'Expanded View' section of the submission guidelines.

- Additional Tables/Datasets should be labeled and referred to as Table EV1, Dataset EV1, etc. Legends have to be provided in a separate tab in case of .xls files. Alternatively, the legend can be supplied as a separate text file (README) and zipped together with the Table/Dataset file.

4) a .docx formatted letter INCLUDING the reviewers' reports and your detailed point-by-point responses to their comments. As part of the EMBO Press transparent editorial process, the point-by-point response is part of the Review Process File (RPF),

which will be published alongside your paper.

5) a complete author checklist, which you can download from our author guidelines. Please insert information in the checklist that is also reflected in the manuscript. The completed author checklist will also be part of the RPF.

6) Please note that all corresponding authors are required to supply an ORCID ID for their name upon submission of a revised manuscript (<https://orcid.org/>), which can be linked to your profile in the manuscript submission system.

7) Before submitting your revision, primary datasets produced in this study need to be deposited in an appropriate public database. Please remember to provide a reviewer password if the datasets are not yet public. The accession numbers and database should be listed in a formal "Data Availability" section placed after Methods. Please note that the Data Availability Section is restricted to new primary data that are part of this study. * Note - All links should resolve to a page where the data can be accessed. *

If your study has not produced novel datasets, please mention this fact in the Data Availability Section.

8) At EMBO Press we ask authors to provide source data for the main manuscript figures. You will receive a separate email with instructions for providing source data with your revised manuscript, including how to upload and organize the files.

9) Our journal also encourages inclusion of *data citations in the reference list* to directly cite datasets that were re-used and obtained from public databases. Data citations in the article text are distinct from normal bibliographical citations and should directly link to the database records from which the data can be accessed. In the main text, data citations are formatted as follows: "Data ref: Smith et al, 2001" or "Data ref: NCBI Sequence Read Archive PRJNA342805, 2017". In the Reference list, data citations must be labeled with "[DATASET]". A data reference must provide the database name, accession number/identifiers and a resolvable link to the landing page from which the data can be accessed at the end of the reference.

10) Regarding data quantification, the following points must be specified in each figure legend:

- the name of the statistical test used to generate error bars and P values,

- the number (n) of independent experiments (please specify technical or biological replicates) underlying each data point,

- the nature of the bars and error bars (s.d., s.e.m.),

- If the data are obtained from n Program fragment delivered error ``Can't locate object method "less" via package "than" (perhaps you forgot to load "than"?) at //ejpvfs23/sites23b/embor_www/letters/embor_decision_revise_and_review.txt line 54.' 2, use scatter blots showing the individual data points.

Discussion of statistical methodology can be reported in the materials and methods section, but figure legends should contain a basic description of n, P and the test applied.

- Please also include scale bars in all microscopy images.

11) The journal requires a statement specifying whether or not authors have competing interests (defined as all potential or actual interests that could be perceived to influence the presentation or interpretation of an article). In case of competing interests, this must be specified in your disclosure statement.

12) All Materials and Methods need to be described in the main text using our 'Structured Methods' format, which is required for all research articles. According to this format, the Methods section includes a Reagents and Tools Table (listing key reagents, experimental models, software and relevant equipment and including their sources and relevant identifiers) followed by a Methods and Protocols section describing the methods using a step-by-step protocol format. The aim is to facilitate adoption of the methodologies across labs. More information on how to adhere to this format as well as a downloadable template (.docx) for the Reagents and Tools Table can be found in our author guidelines.

We would also welcome the submission of cover suggestions, or motifs to be used by our Graphics Illustrator in designing a cover.

As part of the EMBO publication's Transparent Editorial Process, EMBO reports publishes online a Review Process File (RPF) to accompany accepted manuscripts. This File will be published in conjunction with your paper and will include the referee reports, your point-by-point response and all pertinent correspondence relating to the manuscript.

I look forward to seeing a revised form of your manuscript.

Yours sincerely,

Bernd Pulverer

~~~~~~~~~~~~~~~~~~~~~~~~~~~~~~~~~
Bernd Pulverer, Ph.D.
Chief Editor, EMBO Reports
EMBO
Meyerhofstrasse 1, D-69117 Heidelberg
Tel: +4962218891501
bernd.pulverer@embo.org
~~~~~~~~~~~~~~~~~~~~~~~~~~~~~~~~~

Referee #1:

There has been ongoing discussion in the field regarding the utility of the p16-3MR mouse line developed by Demaria et al. (Dev. Cell, 2014). In this manuscript from the Demaria Lab, the authors attempt to address the limitations and shortcomings highlighted in the recent study by the Hara Lab (submitted to EMBO Reports).

The authors emphasise that the p16-3MR model was widely distributed and cite several studies that reported successful use of the line for RFP detection, bioluminescence imaging, and ganciclovir (GCV)-mediated ablation of p16-expressing cells (Table 1). They then proceed to address the specific concerns raised by Hara et al. experimentally in one figure.

A central point of controversy is whether p16-3MR mice can reliably be used for bioluminescence imaging after administration of CTZ-h, the Renilla luciferase substrate. In Figure 1A, the authors present what appears to be higher bioluminescent signal in 18-month-old versus 4-month-old mice; however, no wild-type controls were included. As a result, it is not possible to determine whether these differences reflect reporter activity or background luminescence caused by CTZ-h independently of RLuc. Figure 1B shows similar data in shaved mice, but again only a single wild-type male is included, which is insufficient for meaningful comparison. Figures 1C and 1D also lack appropriate wild-type controls. Skin injury and doxorubicin treatments are performed solely in p16-3MR mice, and only three animals are analysed. Figure 1E further illustrates this issue: only p16-3MR mice injected with vehicle are shown, without any age-matched wild-type mice subjected to the same procedures. The organ-level bioluminescence analysis is also performed exclusively in p16-3MR mice (Figure 1 F), preventing assessment of background signal.

Taken together, the data presented do not adequately refute the findings of the Hara Lab. Two major issues remain unresolved:

Bioluminescent signal specificity
The evidence to date suggest that the bioluminescent signal in p16-3MR mice is largely unrelated to transgene activity, but instead reflects background luminescence generated by CTZ-h through RLuc-independent mechanisms, such as reactions with albumin (Zhao et al., 2004) or superoxide (Bronsart et al., 2016). Additional variables, e.g. hair cycle, pigmentation, effect of doxorubicin on hair loss and sex-dependent differences in skin thickness (Azzi et al, 2005; Amoh et al, 2007), further complicate interpretation of bioluminescence in aged mice, after skin injury, and in other biological contexts.

Interpretation of GCV effects
The authors do not demonstrate that reductions in bioluminescent signal following GCV treatment are caused by ablation of senescent cells. Given that GCV can inhibit the cGAS/STING pathway (Ding et al, 2014; Gong et al, 2022), a key inducer of senescence, it is plausible that reduced senescence markers and SASP factors observed in prior studies may reflect suppression of senescence induction rather than targeted elimination of senescent cells.

In summary, the current manuscript does not provide sufficient evidence to counter the limitations of the p16-3MR model identified by the Hara Lab. The principal concern is the consistent lack of adequate wild-type controls. Without rigorous comparison to properly matched non-transgenic mice, conclusions regarding bioluminescence, reporter specificity, or the effects of GCV and doxorubicin remain insufficiently supported.

A further point worth raising is that the central controversy may not simply be whether the p16-3MR mouse line as originally described was functional, but whether the currently available versions of the line remain functional today. One plausible explanation for the discrepancies between the original publication, subsequent studies, and the recent work from the Hara Lab is the possibility of transgene silencing over successive generations.

Transgene inactivation is a well-documented phenomenon in transgenic mouse lines and can occur through several mechanisms, including epigenetic silencing, methylation of regulatory sequences, or structural rearrangements over time. It is therefore conceivable that the p16-3MR line functioned as intended shortly after its generation, but that progressive inactivation of the transgene has produced the present-day colonies in which reporter activity is minimal or absent.

This possibility may offer a mechanistic explanation for why some earlier studies reported detectable bioluminescent activity, while more recent evaluations, including the present analysis, find little to no functional signal. It also reinforces the need for rigorous validation and the use of appropriate wild-type controls whenever the p16-3MR model is employed.

Referee #2:

This is a reply from Prof. Demaria to concerns raised by the Hara group. In situations where one is unable to reproduce another's results, yet the other party maintains that everything is correct, I can only comment on the experiments presented therein.

The principal conclusion from Hori's work is that "experimental results obtained without the inclusion of WT mice as negative controls... should be interpreted with caution." In this context, all the results shown in Figure 1 lack appropriate age-matched WT controls. Such controls, including multiple animals, ought to be included for all experiments presented. As Hori has emphasised, any condition-dependent changes in the signal could simply reflect background physiological alterations arising from factors such as ageing or exposure to various treatments.

Could the authors clarify the results shown in the lower graph of Figure 1B? The graph indicates that there is no difference between age-matched WT and transgenic males, whereas an increase is observed in females. Does this mean that the reporter line cannot be used in males? Additionally, what is the background level in age-matched WT female controls?

A further point concerns the organ source of the bioluminescent signal. Whole-body images indicate a maximal signal in the abdominal region, potentially consistent with senescent cells in adipose tissue. Yet, upon examination of organs from aged animals, the lungs and spleen exhibit very high signals-something not apparent in the whole-body imaging. This discrepancy is concerning. As previously noted, age-matched WT controls are absent from this analysis, rendering the interpretation of these results rather challenging.

I am not entirely convinced of the utility of Table 1, as all of these studies have already been published. However, if the authors wish to retain it, it would be informative to indicate which studies actually included age-matched wild-type mice for p16 expression analysis and ganciclovir-treated, age-matched wild-type mice for ablation experiments and whether mice were shaved; this should be clearly specified. Upon reviewing the list, it appears that many publications do not include such important controls.

Ref #3 (general comments):

In my quick review of the two abstracts, I can imagine that each author is partially correct. The substrate coelenterazine is a high energy molecule that is catalyzed by albumin and the stress environment associated with inflammation-the molecule is a good maker of oxidative stress and this is likely why they observe signals in mice without the reporter gene when senescence is induced. In addition the reaction produces blue light which is absorbed by hemoglobin and melanin, so the loss of signal can be significant. The loss of signal and the autocatalysis are likely the cause of the problems in these studies.

Response to Referee #1

Comment: Lack of adequate WT controls undermines interpretation.

Response: We agree that inclusion of littermate WT controls is best practice and note that such controls were included in several of our original and subsequent studies, as well as in multiple independent publications. However, reproducing the entire experimental dataset solely to add additional WT cohorts would require extensive new animal experimentation without generating fundamentally new biological insight. In line with EU and Dutch animal-research regulations and the 3Rs principle, we consider such experiments disproportionate. Instead, we have made all new experimental data publicly available as a preprint and revised the manuscript to a text-only technical clarification focused on interpretation rather than data re-presentation.

Comment: GCV effects may reflect suppression of senescence rather than ablation.

Response: This possibility has been considered and discussed explicitly in the revised manuscript. Importantly, most published p16-3MR studies validated senescent-cell reduction using orthogonal markers independent of the transgene, and functional outcomes are observed selectively in transgenic animals.

Response to Referee #2

Comment: Table 1 is unnecessary.

Response: We respectfully disagree. Table 1 documents independent use of the model outside the originating laboratories and directly addresses reproducibility, which is central to the current discussion.

Comment: Organ-specific signal discrepancies.

Response: This issue is addressed textually by discussing optical attenuation, tissue depth, and imaging geometry, without presenting new data in EMBO Reports. References to a recent pre-print from our group are added.

Response to Referee #3

Response: We agree that CTZ-h chemistry, hemoglobin absorption, and oxidative environments influence signal detection. These points are explicitly incorporated into the revised text as technical considerations rather than evidence of model failure.

Dear Dr. Demaria,

Thank you for the submission of your correspondence to our editorial offices.
Thank you also for your patience: as we discussed, we co-reviewed your manuscript with that of Hara and colleagues (same referees). We therefore had to delay processing to synchronize the manuscripts.

You will find the comments of referees 1 and 2 on the revised version enclosed below. Neither referee recommends publication of the current correspondence.

However, since we are in a position to publish a revised manuscript by Hara and colleagues, we are open to publish a short response from your laboratory that conforms to our general standard for refutations and that addresses the referees comments in as far as it is realistic in this context.
While we will not insist on the additional controls requested and while we will allow a citation to the preprinted data in question, we would only be able to publish a redacted commentary.
For expedience, I have attached an edited version of the manuscript that we would be able to accept for co-publication with the manuscript by Hara and colleagues.
Note that we agree with ref 2: 'table 2 is unnecessary', especially as it does not refer to studies with littermate wt controls. It is a general journal policy not to include literature surveys in refutations - as you will agree, similar situations arise also in other contexts and citations have to be directly relevant to specific data or arguments.

Please consider wether you would be interested in including the revised version of
you manuscript in the journal.

Yours sincerely,

Bernd Pulverer

~~~~~~~~~~~~~~~~~~~~~~~~~~~~~~~~
Bernd Pulverer, Ph.D.
Chief Editor, EMBO Reports
EMBO
Meyerhofstrasse 1, D-69117 Heidelberg
Tel: +4962218891501
bernd.pulverer@embo.org
~~~~~~~~~~~~~~~~~~~~~~~~~~~~~~~~

Referee #1:
The authors have not satisfactorily addressed the central concerns raised in my previous review. As previously outlined, the inclusion of appropriate wild-type controls lacking the p16-3MR allele is essential to validate the experimental system and to support the interpretation of the findings. The absence of such controls represents a significant limitation, and no data have been provided to address this point.

Furthermore, the authors do not present convincing evidence that the observed reduction in senescence markers is due to specific ablation of senescent cells. Alternative explanations, including non-specific or indirect effects of ganciclovir treatment, have not been adequately considered or experimentally excluded.

These issues are particularly important in light of the well-documented limitations and concerns surrounding the p16-3MR model, including those highlighted in the work by Hara and colleagues. In the absence of rigorous controls and direct evidence, the conclusions drawn from this model remain insufficiently supported.

Referee #2:
None of my concerns have been experimentally addressed, and I am rather surprised that the authors consider the request to include appropriate controls to be experimentally disproportionate. As noted in my previous review, the absence of proper controls raises serious concerns regarding the overall validity of the model, and, in its current form, this deficiency precludes the publication of the manuscript.
.....

The authors have addressed the editor's requests.

2nd Revision - Editorial Decision 26th Apr 2026

Dear Dr. Demaria

Thank you for the submission of your correspondence to our editorial offices. I have now read it again and we will be glad to publish it pending two minor changes:
1) the following sentence was not in the earlier version and I would like to query it: 'While we were involved in the first publication describing applications of this model, we were not involved in its generation and initial technical validation. Our perspective here is therefore that of experienced users of the system...'. I believe your Dev Cell paper was the first report of the p16-3MR mouse model and since you are the first authors it will confuse the general reader - I would suggest to either delete the comment, as all authors are ultimately responsible for the content of a paper as per standard journal author guidelines, or to highlight specifically a section on author contribution from the paper that explains details (if available).
2) Please also use our specific formatting for preprint citations by adding [PREPRINT] after the Wang B. et al reference in the ref list.

We will proceed with publication as soon as we have a final person in hand.

Yours sincerely,

Bernd Pulverer

~~~~~~~~~~~~~~~~~~~~~~~~~~~~~~~~
Bernd Pulverer, Ph.D.
Chief Editor, EMBO Reports
EMBO
Meyerhofstrasse 1, D-69117 Heidelberg
Tel: +4962218891501
bernd.pulverer@embo.org
~~~~~~~~~~~~~~~~~~~~~~~~~~~~~~~~

The authors have addressed the editor's requests.

Prof. Marco Demaria
University Medical Center Groningen
European Research Institute for the Biology of Ageing
Antonius deusinglaan 1
Groningen, Groningen 9713
Netherlands

Dear Prof. Demaria,

Thank you for the submission of your correspondence to our editorial offices. I have now accepted it for publication.

Your manuscript will be copy edited and you will receive page proofs prior to publication. Please note that you will be contacted by Springer Nature Author Services to complete licensing information.

Please contact me any time of you have any questions.

Yours sincerely

Bernd Pulverer

~~~~~~~~~~~~~~~~~~~~~~~~~~~~~~~~~
Bernd Pulverer, Ph.D.
Chief Editor, EMBO Reports
EMBO
Meyerhofstrasse 1, D-69117 Heidelberg
Tel: +4962218891501
bernd.pulverer@embo.org
~~~~~~~~~~~~~~~~~~~~~~~~~~~~~~~~~
